# Intra-articular injection of micronized dehydrated human amnion/chorion membrane attenuates osteoarthritis development

**DOI:** 10.1186/ar4476

**Published:** 2014-02-06

**Authors:** Nick J Willett, Tanushree Thote, Angela SP Lin, Shamus Moran, Yazdan Raji, Sanjay Sridaran, Hazel Y Stevens, Robert E Guldberg

**Affiliations:** 1 George W. Woodruff School of Mechanical Engineering, Georgia Institute of Technology, 315 Ferst Drive, Atlanta, GA 30332-0405, USA; 2 Parker H. Petit Institute for Bioengineering and Bioscience, Georgia Institute of Technology, 315 Ferst Drive, Atlanta, GA 30332-0405, USA; 3 Wallace H. Coulter Department of Biomedical Engineering, Georgia Institute of Technology, 315 Ferst Drive, Atlanta, GA 30332-0405, USA

## Abstract

**Introduction:**

Micronized dehydrated human amnion/chorion membrane (μ-dHACM) is derived from donated human placentae and has anti-inflammatory, low immunogenic and anti-fibrotic properties. The objective of this study was to quantitatively assess the efficacy of μ-dHACM as a disease modifying intervention in a rat model of osteoarthritis (OA). It was hypothesized that intra-articular injection of μ-dHACM would attenuate OA progression.

**Methods:**

Lewis rats underwent medial meniscal transection (MMT) surgery to induce OA. Twenty four hours post-surgery, μ-dHACM or saline was injected intra-articularly into the rat joint. Naïve rats also received μ-dHACM injections. Microstructural changes in the tibial articular cartilage were assessed using equilibrium partitioning of an ionic contrast agent (EPIC-μCT) at 21 days post-surgery. The joint was also evaluated histologically and synovial fluid was analyzed for inflammatory markers at 3 and 21 days post-surgery.

**Results:**

There was no measured baseline effect of μ-dHACM on cartilage in naïve animals. Histological staining of treated joints showed presence of μ-dHACM in the synovium along with local hypercellularity at 3 and 21 days post-surgery. In MMT animals, development of cartilage lesions at 21 days was prevented and number of partial erosions was significantly reduced by treatment with μ-dHACM. EPIC-μCT analysis quantitatively showed that μ-dHACM reduced proteoglycan loss in MMT animals.

**Conclusions:**

μ-dHACM is rapidly sequestered in the synovial membrane following intra-articular injection and attenuates cartilage degradation in a rat OA model. These data suggest that intra-articular delivery of μ-dHACM may have a therapeutic effect on OA development.

## Introduction

Osteoarthritis (OA) is a degenerative joint disease that is currently the leading cause of chronic disability in the US [1]. Additionally, it is the single most expensive condition among Medicare patients in the US [2]. OA is characterized by chronic degeneration of the articular cartilage through processes such as depletion of proteoglycans (PG), hypertrophic differentiation of chondrocytes, surface erosion, lesion formation and mineralization of the extracellular matrix (ECM) [3,4]. Current treatment options are targeted towards symptomatic management via pain relief drugs or surgical intervention; however, both have clear limitations [4]. Non-steroidal anti-inflammatory drugs (NSAIDs) are the most commonly used class of pain relief drugs but have strong side-effect profiles including gastro-intestinal complications [1]. Grafting and surgical reconstructions on the other hand are associated with poor outcomes and associated pain [4]. Clinical trials have tested a number of potential disease modifying OA drugs (DMOADs), including matrix-metalloproteinase inhibitors (MMPis), cytokine blockers, inhibitors of inducible nitric oxide synthase (iNOS), and doxycycline; however, none have shown a clear therapeutic benefit to date [5]. There are multiple DMOADs in clinical trials, such as rhFGF-18 (Sprimerfin by Merck), a growth factor based therapy, in Phase II which has shown a decrease in cartilage thickness and volume in patients with OA [6]. Strontium ranelate, in a recently completed Phase 3 clinical trial showed a significant improvement in the joint structure of OA patients [7]. Although heavy investment in research continues, OA remains a pervasive and burdensome condition with limited effective clinical options, and there is still a critical need to investigate novel OA therapies.

Testing therapeutics for OA requires selection of a suitable animal model and an efficient and quantitative analysis method. The medial meniscal transection (MMT) rat model is a well- accepted small animal model for evaluating new pharmacologic agents [8]. Although the MMT model is considered a screening model, the predominant analytical method, histology, is a slow, costly, and labor intensive process with poor sensitivity due to the two-dimensional semi-quantitative nature of histopathological scoring [8,9]. In comparison, micro-computed tomography (μCT) imaging can more rapidly and quantitatively evaluate three-dimensional morphologic and degenerative changes in articular cartilage by adaption with a technique relying on the equilibrium partitioning of an ionic contrast agent (EPIC-μCT) [10]. This technique is based on the principle that compared to healthy cartilage degenerated cartilage contains a lower proteoglycan content and, therefore, a higher concentration of a negatively charged contrast agent at equilibrium. Previous studies have validated this methodology to quantify compositional and structural changes, such as cartilage proteoglycan content, thickness and volume [9,11,12]. Our previous study has characterized the rat MMT model using EPIC-μCT and demonstrated changes in the articular cartilage [13]. These studies have further demonstrated the sensitivity to detect chemically- or mechanically-induced degenerative changes. However, EPIC-μCT has not previously been applied to the evaluation of the efficacy of potential OA therapeutics.

Locally delivered ECM proteins are a promising treatment strategy being developed for a wide range of regenerative applications [14]. Dehydrated human amnion/chorion membrane (dHACM) is a tissue derived from donated placentae which possesses anti-inflammatory properties, displays low immunogenicity and promotes wound healing while inhibiting scar formation [15-17]. There is an established precedence of using this tissue for regenerative applications clinically, ranging from corneal defects to tendon repair [18-23].

The potential for dHACM to modulate the development of OA has not been previously investigated; however, indirect evidence suggests that it may have beneficial effects on cartilage. *In vitro*, dHACM has been shown to maintain chondrocyte phenotype when seeded with chondrocytes [24,25]. *In vivo*, chondrocyte seeded dHACM enhanced regeneration of hyaline cartilage in rabbit osteochondral defects after eight weeks [26]. One of the limitations of these previous studies has been the use of dHACM in the form of sheets which can only treat large defects and would require an invasive surgery. Micronized dHACM (μ-dHACM) offers a minimally invasive, injectable therapy with a longer shelf life than cellularized constructs. μ-dHACM can be devitalized while preserving its basement membrane structure and a variety of growth factors, including platelet-derived growth factor (PDGF), fibroblast growth factor (FGF) and transforming growth factor-beta (TGF-β) [27].

An injectable formulation of μ-dHACM (EpiFix^®^ Injectable, MiMedx Group, Inc. Marietta, GA, USA) has recently been developed that retains the active factors of the original tissue and allows for minimally invasive local delivery targeted to the disease site. μ-dHACM is developed after the native amnion undergoes the PURION^®^ process which maintains bioactive components despite the tissue being devitalized and dehydrated (US Patent 8,357,403– Placenta Tissue Grafts. US Patent 8,372,437-Placenta Tissue Grafts.14. US Patent 8,409,626-Placenta Tissue Grafts). The tissue retains the epithelial and chorion layer after the process. A recent study examined the biological properties of μ-dHACM and *in vitro* experiments show quantifiable levels of various growth factors, interleukins and *tissue inhibitors of metalloproteinases *(TIMPS). A subcutaneous *in vivo* experiment in the same study demonstrated recruitment of mesenchymal progenitor cells to the site of implantation as well. The objective of this study was to quantitatively assess the efficacy of μ-dHACM as a disease modifying intervention in the rat MMT model of joint degeneration. It was hypothesized that a single intra-articular injection of μ-dHACM would attenuate OA disease progression in the rat MMT model.

## Methods

### Surgical methods

Weight matched adult male Lewis rats (Charles River, Wilmington, MA, USA) weighing 300 to 325 g, were acclimated for one week. For the MMT surgery, the animals were anesthetized with isoflurane, and the skin over the medial aspect of the left femoro-tibial joint was shaved and aseptically prepared. The medial collateral ligament (MCL) was exposed by blunt dissection and transected to reflect the meniscus toward the femur. The joint space was visualized, and a full thickness cut was made through the meniscus at its narrowest point [28]. The skin was closed with 4.0 Vicryl sutures and then stapled using wound clips. Twenty-four hours post MMT surgery, either μ-dHACM (AmnioFix^®^ Injectable, MiMedx Group Inc. Marietta, GA, USA) or saline was injected intra-articularly into the stifle joint of the left leg (n = 5 per group, per time point). We have previously performed gait analysis on MMT animals at three weeks showing no effect of the surgery on rat ambulatory movements (Additional file 1: Figure S1). The μ-dHACM was sourced from two different donors processed in two different batches. The μ-dHACM was resuspended according to the product insert and injected into the articular joint space using a 25 gauge needle. Naïve animals did not undergo any surgical procedure and were injected with μ-dHACM or saline. The μ-dHACM was manufactured using the proprietary PURION^®^ process and is compliant with the American Association of Tissue Banks regulations for donor tissues (MiMedx Group, Inc. Marietta, GA, USA). This process produces a dehydrated, devitalized amnion and chorion tissue graft which is then micronized, sterilized and can be suspended in saline (US Patent 8,357,403– Placenta Tissue Grafts; US Patent 8,372,437-Placenta Tissue Grafts; US Patent 8,409,626-Placenta Tissue Grafts). Animals were euthanized at three or twenty-one days post-surgery. The Georgia Institute of Technology Institutional Animal Care and Use Committee approved the experimental procedures for these *in vivo* studies (IACUC protocol #A09007).

### Synovial fluid analysis

Rats were euthanized via CO_2_ inhalation at three days or twenty-one days post-surgery for both the naïve group and the MMT surgery group. Synovial fluid was collected by first injecting 100 μl of saline intra-articularly using a 30 gauge insulin syringe, followed by aspirating approximately 50 to 100 μl of the synovial fluid and saline using the same syringe. Synovial fluid was analyzed using the Quantibody^®^ Rat Inflammation Array 1, a multiplex ELISA kit that quantitatively measured 10 rat inflammatory factors: IFNγ, IL-1α, IL-1β, IL-2, IL-4, IL-6, IL-10, IL-13, monocyte chemoattractant protein-1 (MCP-1), and TNFα (RayBiotech, Norcross, GA, USA).

### EPIC-μCT analysis of articular cartilage

Articular cartilage structure and composition were quantitatively evaluated in the tibial plateau as described in our previous study [13]. Scanco evaluation software was used to assess three-dimensional morphological parameters and local attenuation. Dissected tibia were immersion fixed in 10% neutral buffered formalin for 48 hours then stored in 70% ethanol until ready for scanning. Immediately prior to scanning, tibiae were immersed in 2 ml of 30% Hexabrix™ 320 contrast agent (Covidien, Hazelwood, MO, USA) and 70% PBS at 37°C for 30 minutes [9,10]. Samples were then scanned using a μCT 40 (Scanco Medical, Brüttisellen, Switzerland) at 45 kVp, 177 μA, 200 ms integration time, and a voxel size of 16 μm [9]. Raw data were automatically reconstructed to two-dimensional grayscale tomograms. These were rotated to sagittal sections, and the cartilage was contoured and thresholded with global segmentation parameters (Gauss sigma - 1.2, support - 2, threshold - 175 to 225). Direct distance transformation algorithms were used to quantify three-dimensional morphology [9,29,30]. The subsequently generated three-dimensional images were delineated into three volumes of interest (VOIs). For the MMT model, images were evaluated for: 1) full articular cartilage in the proximal tibia including medial and lateral aspects; 2) medial 1/3 of the medial tibial plateau; and 3) focal lesions on the medial plateau only. Outcome measures included average articular cartilage thickness, volume and attenuation. Cartilage attenuation is a quantitative parameter that is inversely proportional to sulfated glycosaminoglycan (sGAG) content [10]. Degraded cartilage contains lower sGAG content and, therefore, higher contrast agent content and higher attenuation values [9]. Two outcome measures were defined for evaluation of focal defects: erosion (defect depth extending to less than 50% of cartilage thickness) and lesion (defect depth extending to more than 50% of cartilage thickness) [31]. To define VOIs of focal lesions, manual contouring was performed in the isolated lesion area to account for the curvature of the surrounding cartilage tissue and exclude the subchondral bone and surrounding air. Within this VOI, segmented cartilage volume was subtracted from the total volume evaluated to compute lesion volume. A summary of the various outcome measures is presented in Table 1.

**Table 1:** Summary of experimental groups and outcome measurements

**Groups**	**Day 3 measurements**	**Day 21 measurements**
μ-dHACM	Histology (full joint)	Histology (femora only)
(naïve rats)	Synovial fluid analysis	Synovial fluid analysis
Control: Saline		Micro-CT
(naïve rats)		
μ-dHACM + MMT rats	Histology (full joint)	Histology (femora + tibiae
Control: Saline +	Synovial fluid analysis	separate)
MMT rats		Synovial fluid analysis
		Micro-CT

CT, computed tomography; MMT, medial meniscal transection; μ-dHACM, micronized dehydrated human amnion/chorion membrane.

### Histology: synovium and cartilage

For the three day time point, the entire limb was harvested with knee joint intact, fixed in 10% neutral buffered formalin for three to four days, transferred to 70% ethanol and stored at 4°C. For the 21 day time point, the tibiae and femora were harvested separately, dissected free of surrounding tissues, fixed in 10% neutral buffer formalin for three to four days, transferred to 70% ethanol and stored at 4°C. For femoral dissections, care was taken to preserve the synovium, meniscus and femoro-meniscal connective tissue. Following EPIC-μCT scanning at 21 days, tibiae and femora were decalcified in Cal-Ex II (Fisher Scientific, Waltham, MA, USA) for 14 days. Dehydrated samples were routinely processed into paraffin embedded blocks. For comparison with EPIC-μCT images, sagittal sections were cut at 5 μm thickness. Sections were stained for sGAGs with a 0.5% Safranin-O (Saf-O) solution and a 0.2% aqueous solution of FastGreen as a counter-stain or with hematoxylin and eosin (H&E).

### Statistical analysis

All quantitative data were expressed as mean ± standard error. Cartilage morphology and composition parameters between groups were evaluated using a one factor (left versus right) analysis of variance (ANOVA) with Tukey’s test for *post-hoc* analysis. Cartilage morphology and composition parameters within the same group were compared between experimental and control knees using paired t-tests. Statistical significance was set at *P* <0.05. All data were analyzed using GraphPad Prism software version 5.0 (GraphPad Software, Inc., La Jolla, CA, USA).

## Results

### μ-dHACM injected into naïve joints

**Figure 1:**
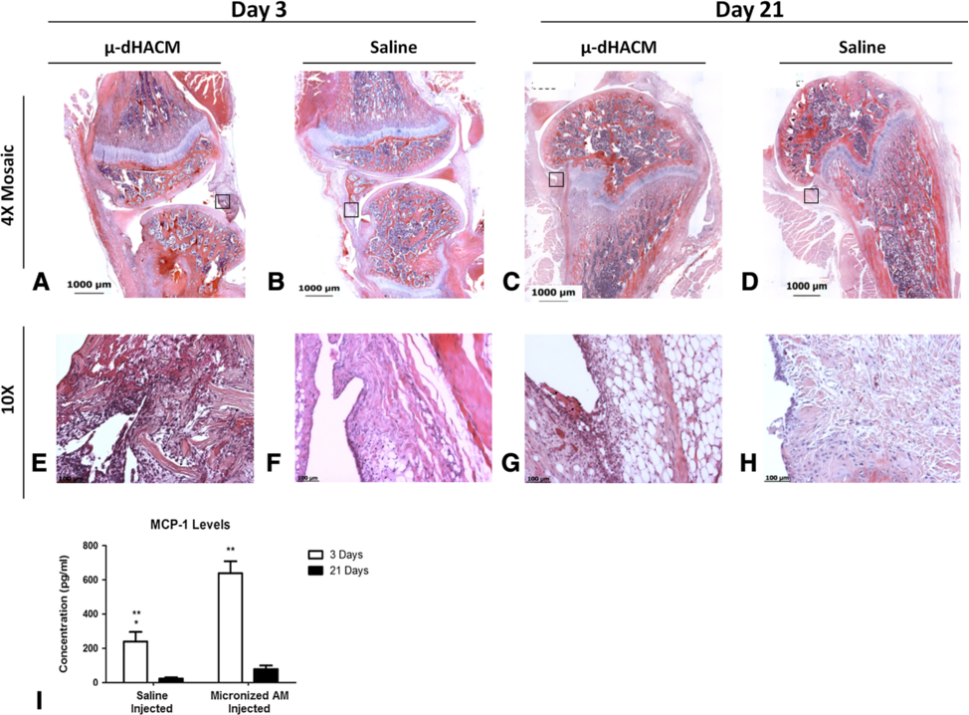
**Representative histology images of μ-dHACM treated joints and MCP-1 data. A-H)** Representative histology images of μ-dHACM treated naïve joints and saline treated naïve joints. **E-H** are zoomed in versions of regions of interest (black box) defined in **A-D**, respectively. μ-dHACM is visible at three days as fibrillar eosinophilic material, as indicated by the black arrows **(E and G)**. Hypercellularity was observed in areas around dHACM fragments. **I)** MCP-1 levels in μ-dHACM treated and saline injected naïve rats. MCP-1 levels were significantly higher at three days in both saline and μ-dHACM injected groups compared to twenty-one days. The MCP-1 level at three days was significantly higher in the μ-dHACM injected joints compared to saline treated joints. **P <0.05 for MCP-1 levels at three and twenty-one days for both groups. *P <0.05 for MCP-1 levels at day three in saline injected and μ-dHACM injected joints. n = 5. MCP-1, monocte chemoattractant protein-1; μ-dHACM, micronized dehydrated human amnion/chorion membrane.

μ-dHACM was injected into naïve joints to assess the inflammatory response at three and twenty-one days. Histology three days post-injection showed μ-dHACM particles incorporated into the adjacent synovial tissue (Figure 1). The material appeared as variably-sized fragments of multi-layered eosinophilic, fibrillar material. dHACM fragments were surrounded by moderate inflammatory infiltrate consisting of macrophages, lymphocytes and plasma cells indicating an inflammatory response to the xenogeneic material. No fragments were observed in control samples injected with saline, and these samples exhibited minimal hemorrhage or inflammatory cell presence in the synovial membrane. At 21 days post-surgery, histological staining of μ-dHACM treated stifle joints showed the continued presence of μ-dHACM in the synovium along with inflammatory cells. At twenty-one days, a distribution of smaller sized dHACM particles appeared compared to the three-day time point and a similar foreign body response was observed surrounding the μ-dHACM fragments at both time points (Figure 1). Synovial fluid showed a significant increase in MCP-1 concentration in μ-dHACM-treated joints compared to control joints at three days but no difference was observed at twenty-one days. MCP-1 levels significantly decreased from day three to day twenty-one in both treated and untreated groups (Figure 1). The other cytokines tested did not show any significant differences or were below the limit of detection. Numerical values for all cytokines are presented in Additional file 2: Table S1.

**Figure 2:**
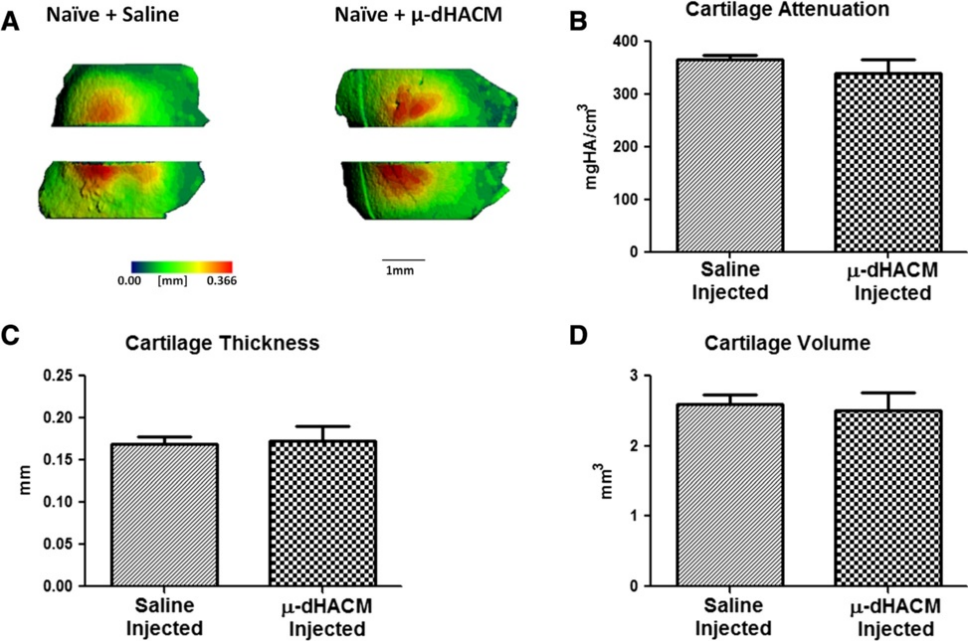
**Effect of μ-dHACM on naïve tibial cartilage. A)** Representative EPIC-μCT tibial articular cartilage thickness maps for μ-dHACM and saline injected samples in naïve joints. **B)** Average cartilage attenuation. **C)** Average cartilage thickness. **D)** Average cartilage volume measure at 21 days. No differences in cartilage parameters were observed between μ-dHACM and saline injected groups. n = 5. EPIC-μCT, equilibrium partitioning of an ionic contrast agent-micro computed tomography; μ-dHACM, micronized dehydrated human amnion/chorion membrane.

Cartilage structure and composition were assessed at 21 days using EPIC-μCT. Representative three-dimensional reconstructions of the cartilage with thickness heat maps appeared normal in structure in naïve animals treated with μ-dHACM or saline (Figure 2). Quantitative assessments of cartilage volume and thickness indicated no differences between saline and μ-dHACM treated joints (Figure 2). Similarly, cartilage attenuation showed no significant difference between saline and μ-dHACM treated joints, indicating no differences in PG content (Figure 2). No lesions or erosions were observed in saline or μ-dHACM injected naïve joints.

### μ-dHACM injected into MMT joints

**Figure 3:**
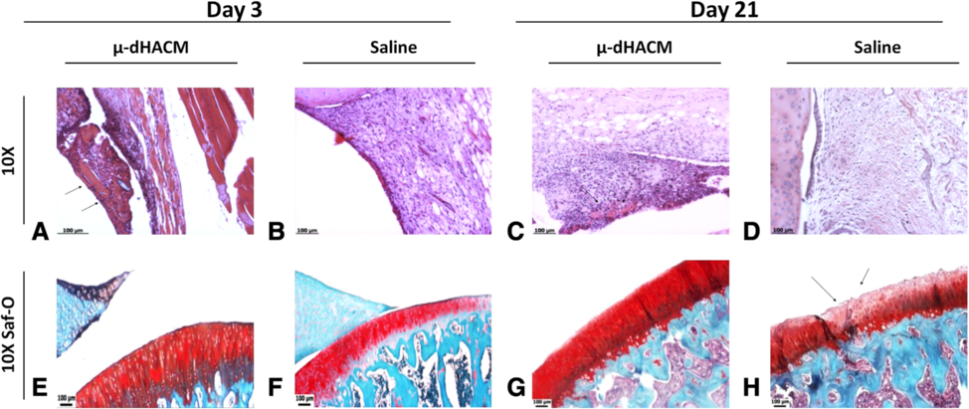
**Effect of μ-dHACM on MMT joints as depicted by histology. A-D)** Representative H & E stained histology images of μ-dHACM treated MMT joints and saline treated MMT joints. μ-dHACM visible at three days as fibrillar eosinophilic material as indicated by the black arrows **(A and C)**. Hypercellularity observed in the area around dHACM fragments. **E-H)** Representative Safranin-O stained histology images of tibia. Black arrows indicate damaged cartilage surface **(H)**. No damage was observed in μ-dHACM treated MMT joints **(G)** whereas erosions and weak staining for PGs were observed in saline treated MMT joints **(H)** at 21 days. MMT, medial meniscal transection; PGs, proteoglycans; μ-dHACM, micronized dehydrated human amnion/chorion membrane.

MMT joints were injected with μ-dHACM or saline, and the effects on the joint and synovium were assessed at three and twenty-one days. Histological analysis showed μ-dHACM incorporated into the synovium at both time points. As observed in naïve joints, the amnion fragments were surrounded by moderate inflammatory infiltrate and appeared reduced in size at 21 days. No fragments were visible in saline treated MMT animals at either three or twenty-one days (Figure 3). In the saline treated MMT animals at 21 days, lesions and areas of erosion were evident along with diminished Saf-O staining of the tibial surface suggesting loss of PG content. In contrast, histological analysis of dHACM treated joints showed a smooth cartilage surface with no lesions and strong Saf-O staining (Figure 3) (Additional file 3: Figure S2 shows additional images). Histological observations did not indicate any differences in cartilage morphology between the two groups at three days. Synovial fluid collected at three and twenty-one days showed no significant differences and most cytokines were below the limit of detection. Numerical values for all cytokines are shown in the Additional file 2: Table S1.

**Figure 4:**
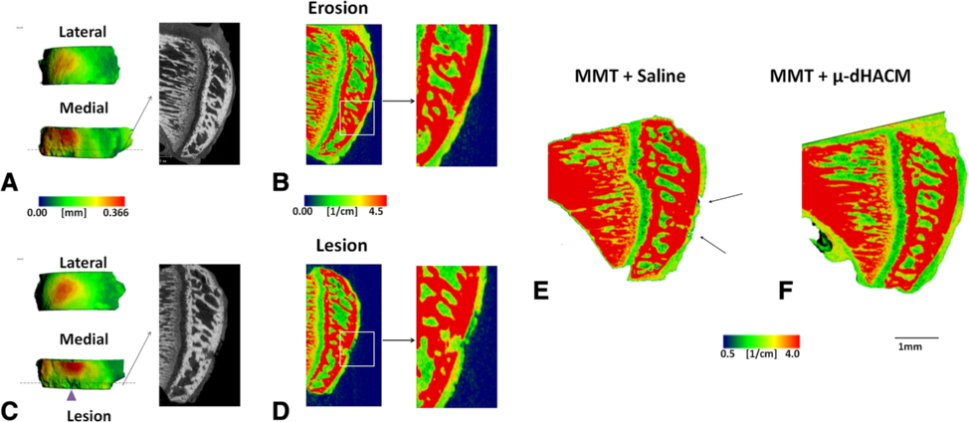
**EPIC-μCT images depicting therapeutic effect of μ-dHACM in MMT joint. A)** Representative image isolating erosion in MMT joint. **B)** Attenuation map indicating erosion on the MMT joint (Inset shows zoomed in view of erosion). **C)** Representative image isolating lesion in MMT joint. **D)** Attenuation map indicating erosion on the MMT joint (Inset shows zoomed in view of erosion). **E-F)** Pseudocolor attenuation map for EPIC-μCT sagittal tibial section for a saline injected **(E)** and dHACM injected **(F)** MMT joint. Red = higher attenuation values (lower PG content), green = lower attenuation values (higher PG content). Black arrows indicate focal defects. The μ-dHACM treated joints did not display lesions on the tibial articular cartilage surface. EPIC-μCT, equilibrium partitioning of an ionic contrast agent-micro computed tomography; MMT, medial meniscal transection; PG, proteoglycans; μ-dHACM, micronized dehydrated human amnion/chorion membrane.

**Figure 5:**
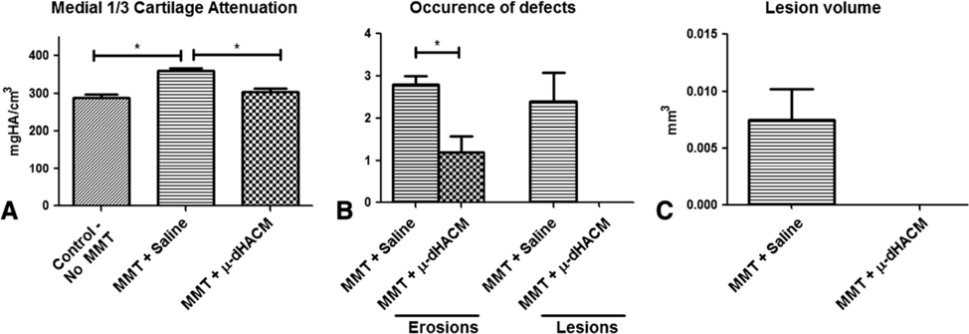
**Quantitative EPIC-μCT data depicting the therapeutic effect of μ-dHACM in MMT joints compared to controls. A)** Average cartilage attenuation was significantly decreased in μ-dHACM treated MMT joints compared to saline injected MMT joints for the medial one-third tibial plateau. **B)** Average number of erosions was significantly decreased in μ-dHACM treated MMT joints compared to saline injected MMT joints. No lesions were observed in the μ-dHACM treated MMT joints. **C)** Average focal lesion volume in saline injected MMT joints.* p <0.05 and n =5. EPIC-μCT, equilibrium partitioning of an ionic contrast agent-micro computed tomography; MMT, medial meniscal transection; μ-dHACM, micronized dehydrated human amnion/chorion membrane.

EPIC-μCT was used to characterize cartilage and focal defects at 21 days (Figure 4). Representative planes and two-dimensional EPIC-μCT attenuation heat maps of the tibial section from μ-dHACM treated MMT joints and saline injected MMT joints are presented (Figure 4). Qualitatively, MMT animals with saline injections displayed noticeable cartilage focal lesions and erosion sites. In contrast, cartilage surfaces in μ-dHACM treated joints appeared more consistently uniform, and no lesions were observed. Quantitative EPIC-μCT at 21 days post-surgery showed that μ-dHACM treated joints had lower cartilage attenuation, indicating higher PG content, in the medial one-third of the tibial plateau compared to saline injected MMT joints. There was no significant difference between average attenuation values between naïve joints and μ-dHACM-treated MMT joints, suggesting the treatment maintained PG content comparable to intact joints (Figure 5). MMT joints injected with saline had an average incidence of 2.8 ± 0.2 erosion and 2.4 ± 0.4 lesion sites per medial tibial plateau with an average lesion volume of .00725 ± .005 mm^3^. μ-dHACM treated MMT joints displayed significantly fewer erosion sites (1.2 ± .374) and no lesions (Figure 5).

## Discussion

There is an unmet medical need for OA therapeutics as no DMOADs have been approved for clinical use. ECMs such as dHACM are a promising therapeutic strategy due to their mix of ECM proteins, growth factors and anti-inflammatory factors [32]. Human dHACM allografts also have an established precedence in clinical applications ranging from corneal defects to tendon repair [18,21]. The objective of this study was to quantitatively assess the efficacy of μ-dHACM (EpiFix^®^ Injectable) as a disease modifying intervention in a rat model of OA.

EPIC-μCT image analysis provided three-dimensional, non-destructive and quantitative evaluation of changes in articular cartilage composition and morphology. The EPIC-μCT data showed a significant reduction in cartilage damage in the form of higher PG levels (lower average attenuation), fewer incidences of erosions and no lesions in the MMT joints treated with μ-dHACM. As histology preparation and sectioning are time-consuming, counting each lesion and erosion would be extremely challenging, and lesion volume would be incalculable given standard two-dimensional metrics. Additionally, traditional histological staining only enables assessment of the cartilage based on a subjective and semi-quantitative scoring system, as previously used to screen DMOADs [9,11]. We also performed histology in this study to compare to our EPIC-μCT results by embedding and processing samples in the sagittal plane. This led to a limitation that the sections could not be graded by traditional methods as those require sectioning in the coronal plane. One study tested MMP inhibitors in the MMT model using outcome measures based on both semi-quantitative scoring and two-dimensional measurements for width and depth of cartilage damage. Due to the inherent variability in these outcome measures, large sample sizes were required to detect the efficacy of this therapy (20 animals per group) [33]. In contrast, in this study we present the EPIC-μCT technique which allowed objective quantification of three-dimensional measures with five animals per group, suggesting that these three-dimensional analyses can provide greater sensitivity for detection of therapeutic effects. EPIC-μCT thus provides a higher throughput method with improved predictive capacity to test the effects of DMOADs in pre-clinical models. Moreover, this is the first study to demonstrate the protective effect of a single intra-articular injection of μ-dHACM in the rat MMT OA model.

There are several possible mechanisms by which dHACM may modulate the progression of OA. A recent study used ELISA assays to analyze growth factor levels present in μ-dHACM and showed quantifiable levels of PDGF-AA, PDGF-BB, TGFα, TGFβ, basic fibroblast growth factor (bFGF), epidermal growth factor (eGF), placental frowth factor (PLGF), granulocyte colony stimulating growth factor, IL-4, IL-6,IL-8, IL-10 and TIMP 1, 2 and 4. The PURION process allowed μ-dHACM to retain its biological activity which may play a role in its effect on attenuation of OA in the rat MMT model. dHACM has been shown to suppress the expression of potent pro-inflammatory cytokines, such as IL-1α and IL-1β, and also to decrease MMP levels through expression of natural MMP inhibitors present in the membrane [34,35]. dHACM also contains IL-1Ra, a receptor antagonist for IL-1, a pro-inflammatory cytokine that has been shown to be upregulated in OA [17]. Two low molecular mass elastase inhibitors – secretory leukocyte proteinase inhibitor (SLPI) and elafin – are expressed in the dHACM, both of which exhibit anti-inflammatory properties [14]. Hyaluronic acid is also present and acts as a ligand for the CD44 receptor which is expressed on inflammatory cells and plays a role in adhesion capabilities of immune cells [14]. In addition to anti-inflammatory molecules, devitalized μ-dHACM particles have been shown to retain a variety of bioactive substances including multiple growth factors such as PDGF and FGF [27]. PDGF and FGF-18 have previously been implicated in chondrocyte growth [36]. All of these potential mechanisms, including maintenance of cartilage homeostasis and reduction of inflammation and MMP expression, may be active contributing factors to the observed protective effect of μ-dHACM. dHACM also retains natural structural components, such as collagens (types I, III, IV, V, VI), fibrinogen, laminin, nidogen and PGs. While these components clearly are important for the regulation and maintenance of normal chondrocyte metabolism, whether the ECM composition of dHACM directly affects OA is unclear [26]. However, retention of the active regulatory components may be influenced by the size and composition of the dHACM particles.

While the mode of action of a disease modifying therapy is a key component of its efficacy, local retention of the factor is also thought to be critical. Retention is particularly challenging when considering small molecule DMOADs. The particle size of the drug or carrier is critical since particles less than 6 microns are quickly filtered through intercellular gaps or phagocytosed by macrophages [37]. Clinical studies measuring residence time of various NSAIDs delivered intra-articularly found the half-life to range from 1.1 to 5.2 hours [38]. Alternatively, larger particles likely elicit an immune response or may cause physical damage to the articular surface [5]. In this study, the μ-dHACM particles sequestered in the synovial membrane at three days were large enough to prevent rapid clearance. Recent research has focused on extending the residence time of drugs in the joint cavity via the use of biomaterials including poly-lactic glycolic acid (PLGA), albumin and bio-polymer based carriers. In pre-clinical models, drug release from PLGA particles has been reported to last up to 14 days, but once released, the drugs were rapidly cleared [39]. Although further studies are needed to elucidate release kinetics and assess whether extended residence time translates to increased duration of therapeutic activity, μ-dHACM presents a potential advantage over other intra-articular therapeutics due to its retention through 21 days in the synovium, although release kinetics of any active factors would also play a role. dHACM thus has the potential to require less-frequent injections to treat OA.

μ-dHACM had not previously been injected into the joint space; therefore, biocompatibility and inflammatory responses were evaluated. Histological analysis indicated moderate local hypercellularity at three days which persisted at twenty-one days. This response was localized to regions surrounding the μ-dHACM particles and was not present throughout the synovial membrane. This agreed with synovial fluid analyses, which demonstrated an increase in MCP-1 for animals treated with μ-dHACM at three days which was then reduced by twenty-one days. This increase in MCP-1 in response to μ-dHACM was not observed in the MMT/saline animals. This could be due to the fact that the MMT surgery itself caused inflammation and masked the upregulation of MCP-1 caused by μ-dHACM treatment. While MCP-1 can activate and recruit monocytes during inflammation, this pro-inflammatory state may even support a healing response depending on the types of monocytes/macrophages recruited [40,41]. We did not observe an upregulation in other pro-inflammatory cytokines. The initial analysis of the inflammatory response has some limitations as levels of most cytokines were below the limit of detection by the ELISA multiplex array. This is likely a technical limitation due to the challenge of harvesting rat synovial fluid, sensitivity of the synovial fluid assay and the limited volume of fluid present in the joint cavity. Alternative methods to explore in the future for synovial fluid analysis include using filter paper absorbent to extract synovial fluid from the joint space and using a more sensitive ELISA such as magnetic bead-based multiplex assays. Another reason for these aberrations could be due to fact that μ-dHACM is a xenograft that is being injected in the rat joint. Although we observed a mild to moderate mononuclear inflammation, we did not identify granulocytes, which would indicate a chronic inflammatory response. Further work is needed to characterize fully the cellular components of the synovial fluid, presence of anti-inflammatory factors as these were observed in *in vitro* studies performed by Koob *et al*. and determine the pathways by which the inflammatory responses progress.

This is a preliminary study testing the effects of dHACM in a rat OA model. There are several areas that need future investigation including greater characterization of the dHACM material. The role of cytokines and growth factors needs to be elucidated in further detail as well. Additional areas of investigation include the effects of particle size, composition of dHACM, timing of injection (pre-existing OA) and duration of particle residence.

## Conclusions

This is the first study demonstrating reduction in cartilage degeneration in a small animal OA model via a single intra-articular injection of a devitalized allograft derived from amniotic membrane. EPIC-μCT, used as an analytical tool, showed, by way of several quantitative metrics, a cartilage-protective role for μ-dHACM. Based on the ability of μ-dHACM to incorporate into the synovial tissue and maintain residency for at least 21 days, this could become a low frequency injection therapeutic. dHACM is a minimally manipulated and marketed allograft material which has been shown to be clinically effective in a variety of applications and now, through this study, μ-dHACM is presented as a candidate to further investigate as a potential disease modifying OA therapy [42,43].

## Abbreviations

μCT: Micro-computed tomography; μ-dHACM: Micronized dehydrated human amnion/chorion membrane; dHACM: Dehydrated human amnion/chorion membrane; DMOADs: Disease modifying osteoarthritis drugs; ECM: Extracellular matrix; ELISA: Enzyme-linked immunosorbent assay; EPIC-μCT: Equilibrium partitioning of an ionic contrast agent; FGF: Fibroblast growth factor; IFN: Interferon; IL: Interleukin; iNOS: Inducible nitric oxide synthase; MCL: Medial collateral ligament; MCP-1: Monocyte chemoattractant protein-1; MMPis: Matrix-metalloproteinase inhibitors; MMT: Medial meniscal transection; NSAIDs: Non-steroidal anti-inflammatory drugs; PBS: Phosphate-buffered saline; PDGF: Platelet-derived growth factor; PG: Proteoglycan; sGAG: Sulfated glycosaminoglycan; SLPI: Secretory leukocyte proteinase inhibitor; Saf-O: Safranin-O; TGF: Transforming growth factor; TIMPs: *Tissue inhibitors of metalloproteinases*; TNFα: Tumor necrosis factor alpha; VOIs: Volumes of interest.

## Competing interests

A gift from MiMedx Group, Inc., Marietta, GA was used to fund this study. MiMedx Group, Inc. Marietta, GA provided EpiFix^®^ Injectable. Robert Guldberg serves on the Medical Advisory Board of MiMedx Group, Inc. and owns stock options in the company. The other authors declare that they have no competing interests.

## Authors' contributions

TT, NJW, AL, HS and RG contributed to the conception and design of the study. The draft manuscript was written by TT and critically revised by all authors. YR and SS performed histology experiments and SM assisted with μCT experiments. RG, AL and HS assisted with manuscript editing and preparation. All authors read and approved the final manuscript.

## Supplementary Material

Additional file 1: Figure S1 Gait analysis data. **A)** Duty cycle. **B)** Print area. Baseline = naïve rats prior to surgery. No differences in duty cycle or print area were observed in MMT rats compared to baseline. n = 8. Click here for file

Additional file 2: Table S1 Cytokine ELISA data with standard deviation - indicates cytokine content lower than limit of detection. Click here for file

Additional file 3: Figure S2 **A-C)** 4X Mosaic representative histology images (H & E) of μ-dHACM treated MMT joints. No lesions are observed on the surface. **D-F)** Zoomed in 10X images show cartilage surface. Only one sample showed surface erosions. **G-I)** 4X Mosaic representative histology images (H & E) of saline treated MMT joints. Lesion on sample is indicated by green arrow **(I). J-L)** Zoomed in 10X images show cartilage surface. Saline treated samples display qualitatively greater surface degeneration. (Black boxes indicated 10X zoomed in areas). Click here for file
