# Supplementary figures and images for: Intra-articular injection of micronized dehydrated human amnion/chorion membrane attenuates osteoarthritis development
Source: Arthritis Res Ther. 2014 Feb 6;16(1):R47. doi: 10.1186/ar4476 (PMC3978824; doi:10.1186/ar4476)

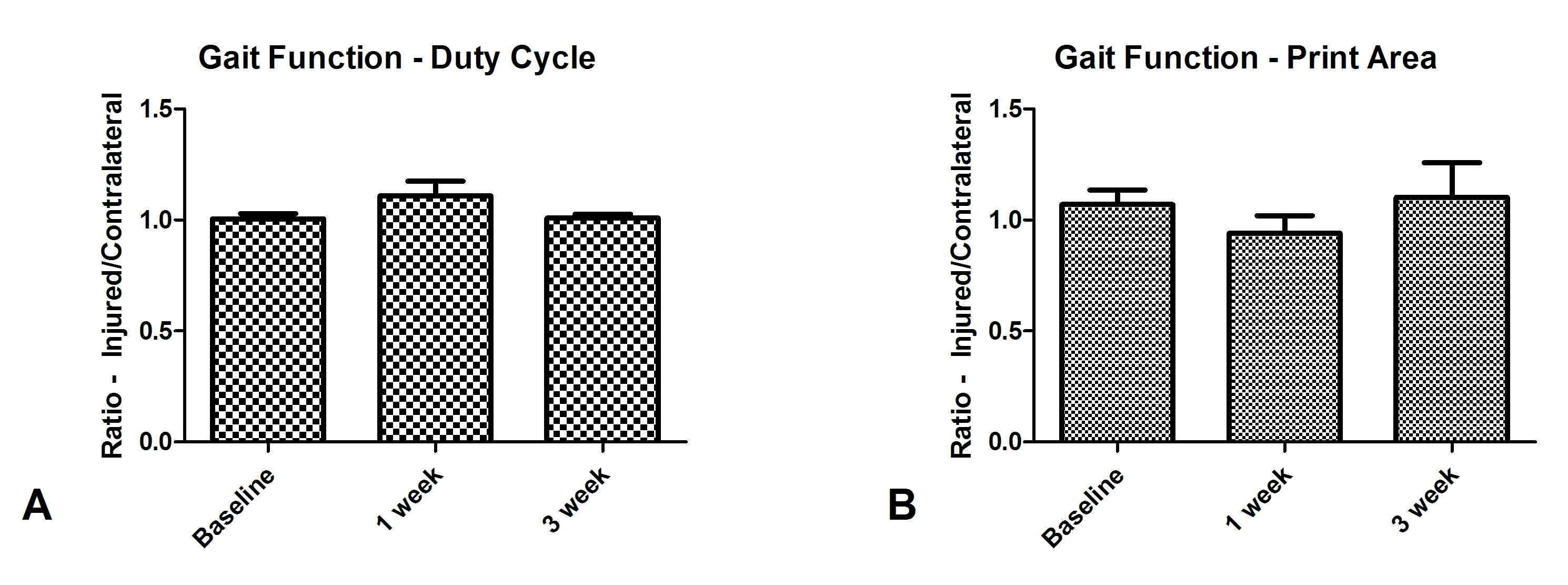

Supplement: Additional file 1: Figure S1 — Gait analysis data. A) Duty cycle. B) Print area. Baseline = naïve rats prior to surgery. No differences in duty cycle or print area were observed in MMT rats compared to baseline. n = 8. [file ar4476-S1.tiff]

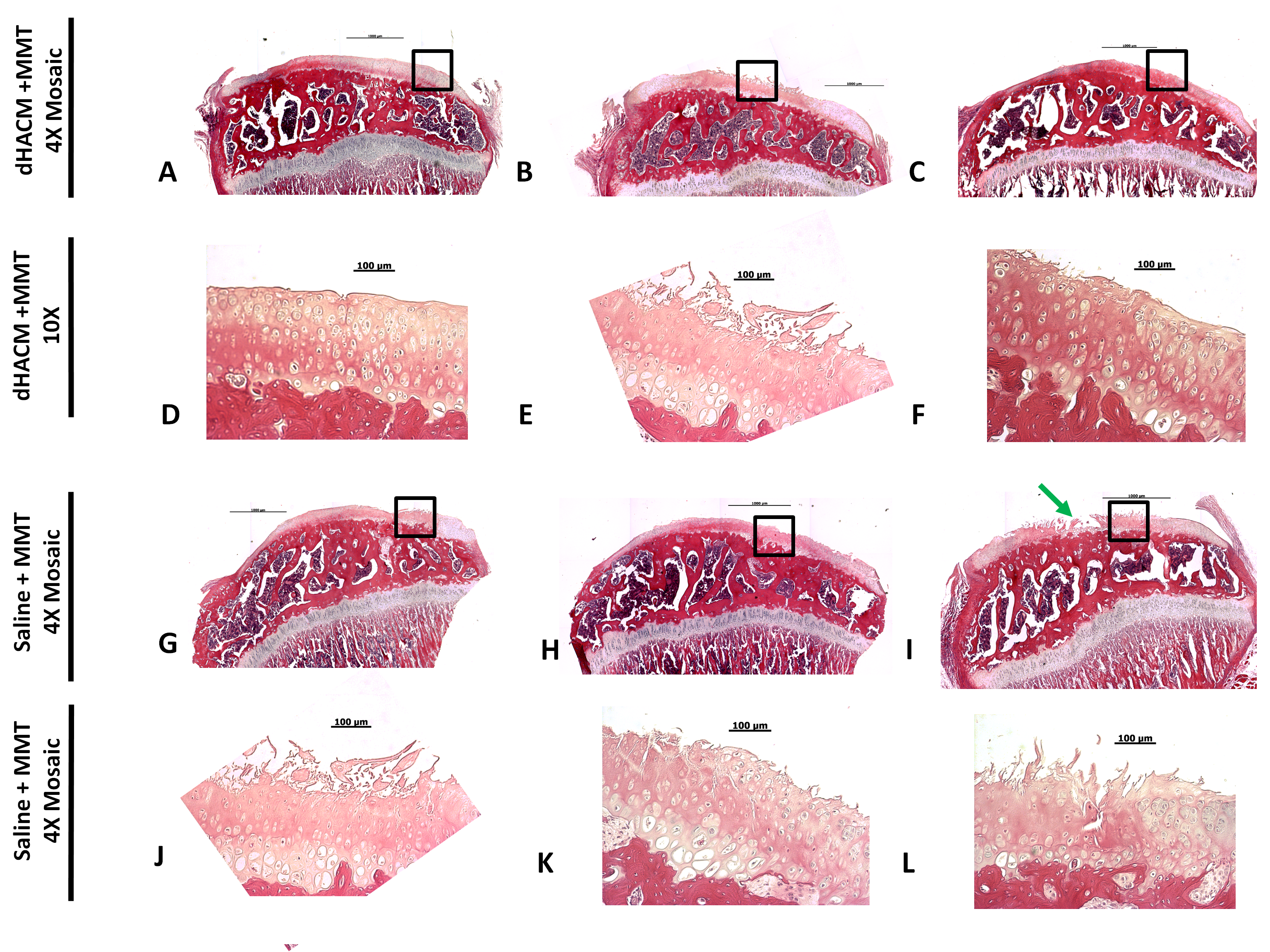

Supplement: Additional file 3: Figure S2 — A-C) 4X Mosaic representative histology images (H & E) of μ-dHACM treated MMT joints. No lesions are observed on the surface. D-F) Zoomed in 10X images show cartilage surface. Only one sample showed surface erosions. G-I) 4X Mosaic representative histology images (H & E) of saline treated MMT joints. Lesion on sample is indicated by green arrow (I). J-L) Zoomed in 10X images show cartilage surface. Saline treated samples display qualitatively greater surface degeneration. (Black boxes indicated 10X zoomed in areas). [file ar4476-S3.tiff]
