# Supplementary material for: Intra-articular injection of micronized dehydrated human amnion/chorion membrane attenuates osteoarthritis development
Source: Arthritis Res Ther. 2014 Feb 6;16(1):R47. doi: 10.1186/ar4476 (PMC3978824; doi:10.1186/ar4476)
Supplement: Additional file 2: Table S1 — Cytokine ELISA data with standard deviation - indicates cytokine content lower than limit of detection. [file ar4476-S2.docx]

| **Cytokines** | **Naïve joints (pg/ml)** | | | | **MMT joints(pg/ml)** | | | |
| --- | --- | --- | --- | --- | --- | --- | --- | --- |
|  | **Day 3** | | **Day 21** | | **Day 3** | | **Day 21** | |
|  | **Saline** | **µdHACM** | **Saline** | **µdHACM** | **Saline** | **µdHACM** | **Saline** | **µdHACM** |
| **IFNγ** | - | - | - | - | - | - | - | - |
| **IL-1a** | - | - | - | - | - | - | - | - |
| **IL-1b** | 46.26  ± 20.42 | 58.35  ± 27.10 | - | - | - | - | - | - |
| **IL-2** | - | - | - | 153.85  ± 113.00 | - | - | - | - |
| **IL-4** | - | - | - | - | - | - | - | - |
| **IL-6** | 60.91  ± 38.54 | 195.87  ± 214.80 | 503.21  ± 178.94 | 253.12  ± 181.61 | - | - | - | - |
| **IL-10** | - | - | - | - | 76.19  ± 27.66 | 69.13  ± 81.83 | - | - |
| **IL-13** | - | - | 61.94  ± 58.28 | - | - | - | - | - |
| **MCP-1** | 239.06  ± 125.48 | 638.16  ± 156.80 | 24.34  ± 16.19 | 79.84  ± 46.35 | 331.31  ± 260.01 | 444.86  ± 200.23 | 102.46  ± 42.10 | 132.09  ± 113.40 |
| **TNFα** | - | - | - | - | 137.44  ± 113.30 | 25.83  ± 44.74 | 117.11  ± 108.90 | 95.65  ± 76.00 |

**Table 2: Cytokine ELISA data with std. dev. - indicates cytokine content lower than limit of detection**
